# Novel predictors of immune checkpoint inhibitor response and prognosis in advanced non‐small‐cell lung cancer with bone metastasis

**DOI:** 10.1002/cam4.5952

**Published:** 2023-04-19

**Authors:** Yohei Asano, Norio Yamamoto, Satoru Demura, Katsuhiro Hayashi, Akihiko Takeuchi, Satoshi Kato, Shinji Miwa, Kentaro Igarashi, Takashi Higuchi, Yuta Taniguchi, Sei Morinaga, Takashi Sone, Miho Okuda, Isao Matsumoto, Seiji Yano, Hiroyuki Tsuchiya

**Affiliations:** ^1^ Department of Orthopaedic Surgery Kanazawa University Graduate School of Medical Sciences Kanazawa Japan; ^2^ Department of Respiratory Medicine Kanazawa University Hospital Kanazawa Japan; ^3^ Department of Respiratory Medicine Ishikawa Prefectural Central Hospital Kanazawa Japan; ^4^ Department of Radiology Kanazawa University Graduate School of Medical Sciences Kanazawa Japan; ^5^ Department of Thoracic Surgery Kanazawa University Kanazawa Japan

**Keywords:** bone metastasis, denosumab, immune checkpoint inhibitor, neutrophil‐to‐lymphocyte ratio, non‐small‐cell lung cancer

## Abstract

**Backgrounds:**

Immune checkpoint inhibitors (ICIs) can significantly prolong the survival of patients with advanced non‐small‐cell lung cancer (NSCLC); however, few studies on the therapeutic effects of ICIs on bone metastases were performed.

**Methods:**

This retrospective study aimed to investigate the therapeutic effects of ICIs and determine predictors of favorable ICI response and prognosis in 55 advanced NSCLC patients with bone metastases who initiated ICI treatment between 2016 and 2019, with a mean follow‐up period of 23.2 months. Patients were classified into responders (complete or partial response) and non‐responders (stable or progressive disease) according to the MD Anderson Cancer Center (MDA) criteria, and the predictors of therapeutic response were identified using multivariate logistic regression analysis. Furthermore, overall survival from the time of ICI administration to the final follow‐up or death was evaluated, and prognostic predictors were identified using Cox proportional hazards regression analysis.

**Results:**

ICI response rate was 30.9% (complete in three cases, partial in 14). Median survival time was 9.3 months, with 1‐year and 2‐year survival rates of 40.6% and 19.3%, respectively. Responders survived significantly longer than non‐responders (*p* = 0.03). Based on the receiver operating characteristic curve, the predictive cutoff value of the pretreatment neutrophil‐to‐lymphocyte ratio (NLR) was 2.1. Multivariate analysis identified female sex (*p* = 0.03), use of ICIs as first‐line therapy (*p* < 0.01), and NLR <2.1 (*p* = 0.03) as significant predictors of therapeutic response, whereas concomitant use of a bone‐modifying agent (*p* < 0.01), Katagiri score ≤6 points (*p* < 0.01), and NLR <2.1 (*p* = 0.02) were identified as significant predictors of good prognosis.

**Conclusions:**

This study identified some novel predictors for favorable therapeutic response and prognosis in advanced NSCLC patients with bone metastases undergoing ICI treatment. Pretreatment NLR less than 2.1 can be considered the most important predictor.

## 1 INTRODUCTION

Lung cancer is one of the most common malignant tumors, with high morbidity and mortality rates worldwide.^1^ Multidisciplinary therapy including chemotherapy, radiation therapy, and surgery has improved the survival time of patients with non‐small‐cell lung cancer (NSCLC), which accounts for 80–85% of primary lung cancers. However, the clinical outcomes are unsatisfactory, and the 5‐year survival rate remains poor.^2^, ^3^, ^4^ Bone metastases are common in lung cancer, with an incidence of 20–40% in NSCLC,^5^, ^6^, ^7^ comparable to that seen in prostate and breast cancer.^8^, ^9^ In 30–60% of such cases,^8^ bone metastases can cause skeletal‐related events (SREs), such as severe bone pain, spinal cord compression, pathological fractures, and hypercalcemia.^10^ Furthermore, SREs significantly reduce a patient's quality of life and lead to a poor prognosis.^10^, ^11^ Therefore, bone metastasis is regarded as an indicator of poor prognosis for lung cancer,^12^, ^13^, ^14^ and appropriate management is required to prevent SREs and improve clinical outcomes.

In recent years, immunotherapy, including the use of immune checkpoint inhibitors (ICIs), has made remarkable progress and has had dramatic therapeutic effects on various malignant tumors.^15^, ^16^, ^17^, ^18^ In NSCLC, ICIs targeting the programmed cell death protein 1 (PD‐1) / programmed death ligand 1 (PD‐L1) pathway, such as PD‐1 inhibitors (nivolumab and pembrolizumab) and PD‐L1 inhibitors (atezolizumab), have significantly prolonged the overall survival (OS) and progression‐free survival (PFS) compared with those of conventional anticancer drugs.^19^, ^20^, ^21^, ^22^ Currently, ICI therapy, either as a monotherapy or combined with platinum‐based chemotherapy, is approved as the standard treatment for advanced NSCLC.^17^

However, considering the poor prognosis of NSCLC patients with bone metastases, the therapeutic effectiveness of ICIs in these patients remains unclear, with few previous reports. Although we have conducted two previous studies on this topic, which revealed favorable therapeutic effects and suggested a new ICI‐based treatment strategy for these patients, they were limited by their small sample size.^23^, ^24^ Therefore, to improve the clinical outcomes of NSCLC patients with bone metastases, further studies are needed to evaluate the therapeutic effectiveness of ICIs in these patients and to identify factors that predict it. Previous studies have reported that factors such as PD‐L1 expression, neutrophil‐to‐lymphocyte ratio (NLR), tumor mutational burden (TMB), and tumor‐infiltrating lymphocytes can indicate the status of the tumor immune microenvironment and act as predictive biomarkers of ICI response.^25^, ^26^, ^27^, ^28^, ^29^ However, no confirmed predictors of ICI response have been identified in patients with bone metastases. Therefore, this study was conducted with the primary purpose of investigating the effects and prognostic outcome of ICI therapy in advanced NSCLC patients with bone metastases, as well as the secondary purpose of identifying predictors of response to therapy and prognostic outcome.

## 2 MATERIALS AND METHODS

### 2.1 Study population

This retrospective study included patients with advanced NSCLC who received ICI treatment between 2016 and 2019 at Kanazawa University Hospital. Patients were included if they were diagnosed with monostotic or multiple bone metastases before the initiation of ICI treatment and were followed up for at least 6 months. The ICIs used were the PD‐1 inhibitors nivolumab and pembrolizumab and the PD‐L1 inhibitors atezolizumab and durvalumab. Pembrolizumab was used as the first‐line therapy in some cases with driver‐gene mutation was negative and a PD‐L1 tumor proportion score (TPS) of ≥50%, while other ICIs were used as second‐line therapy for cases in which treatment with conventional anticancer drugs or molecular‐targeted drugs was ineffective. Cases in which the first‐line therapy consisted of ICIs combined with platinum‐based chemotherapy were also included. Cases in which both PD‐1 and PD‐L1 inhibitors were administered, or a PD‐1/PDL1 inhibitor was combined with a CTLA‐4 inhibitor, were excluded from this study. Moreover, cases who had infectious diseases or other causes of an elevated inflammatory response at the time of pretreatment blood sampling were also excluded. This study was approved by the ethics committee of Kanazawa University Hospital (no. 2019–323), and it conforms to the provisions of the Declaration of Helsinki, and informed consent was obtained from all patients.

### 2.2 Data analysis

Information regarding patient characteristics and treatment history was collected from the database of Kanazawa University Hospital. Clinical data were collected regarding sex, age, histology, PD‐L1 TPS, number of bone metastases, other organ metastases, Eastern Cooperative Oncology Group performance status (ECOG PS), Katagiri score,^30^ pretreatment blood sampling, concomitant use of bone‐modifying agent (BMA), and the line of therapy for which ICIs were administered, and these variables were analyzed as potential predictors of ICI therapy outcome. Blood samples were collected a week prior to ICI treatment initiation, and baseline data were obtained regarding white blood cell (WBC) count, absolute neutrophil count (ANC), absolute lymphocyte count (ALC), platelet count, serum hemoglobin, lactate dehydrogenase (LDH), C‐reactive protein (CRP), creatinine, albumin, alkaline phosphatase (ALP), and the tumor markers CEA, CYFRA, and KL‐6. Furthermore, NLR and platelet‐to‐lymphocyte ratio (PLR) were defined as the ratios of ANC to ALC and platelet count to ALC, respectively, and cutoff values for these variables were determined using receiver operating characteristic (ROC) curves.

The therapeutic effects of ICIs on patients with bone metastases were evaluated using the MD Anderson Cancer Center (MDA) criteria,^31^ and the prognosis was evaluated based on OS from the time of ICI administration to the final follow‐up or death. Based on the MDA criteria, patients were classified into responders (complete response [CR] or partial response [PR]) and non‐responders (stable disease [SD] or progressive disease [PD]), and univariate and multivariate analyses were performed to investigate the predictors associated with therapeutic response and prognosis.

### 2.3 Statistical analysis

The Mann–Whitney *U*‐test and Fisher's exact test were performed to evaluate relationships between clinical variables and the therapeutic effect of ICI treatment. The clinical variables were subjected to univariate analysis with the responder group as reference, and all variables (*p* < 0.05) were subsequently subjected to multivariate logistic regression analysis to identify the best predictors of response to ICI therapy. Furthermore, OS was evaluated using Kaplan–Meier analysis, and the log‐rank test was used to identify variables indicating favorable prognosis. To further clarify the predictors of prognosis, these variables (*p* < 0.05) were subjected to multivariate analysis using the Cox proportional hazards model. Statistical significance was set at a *p*‐value less than 0.05. All statistical analyses were performed using EZR software (Saitama Medical Center, Jichi Medical University, Saitama, Japan).^32^

## 3 RESULTS

### 3.1 Patient characteristics

This study included 55 advanced NSCLC patients with bone metastases, comprising 40 men and 15 women (mean age 66.3 ± 7.9 years), with a mean follow‐up period of 23.2 months. Patient characteristics are summarized in Table 1. The disease stage of 52 patients (94.5%) were IV and 3 (5.5%) were IIIB. Forty‐three patients (78.2%) had multiple bone metastases, and 46 (83.6%) had metastasis in another organ. The most common histology was adenocarcinoma (78.2%), and driver‐gene mutations were observed in 9 patients (8 EGFR+, 1 ALK+). The most commonly used ICI was pembrolizumab (49.1%), which was initiated as first‐line therapy in 15 patients (55.6%). The cutoff values of NLR and PLR determined by ROC curve analysis were 2.1 and 197, respectively.

**TABLE 1:** Characteristics of patients.

	All patients (*n* = 55)	CR + PR (*n* = 17)	SD + PD (*n* = 38)	*p*‐value
Sex				
Male	40	8	32	0.007
Female	15	9	6	
Age	66.3 ± 7.9	67.5 ± 7.8	65.8 ± 7.8	0.457
Histology				
Adenocarcinoma	43	15	28	0.261
Squamous cell carcinoma	7	1	6	
Others	5	1	4	
Driver‐gene mutation				
EGFR	8	4	4	0.379
ALK	1	0	1	
PD‐L1 TPS				
<49%	18	6	12	0.751
≥50%	10	4	6	
Unevaluable	27	7	20	
ICIs				
Nivolumab	15	1	14	0.006
Pembrolizumab	27	9	18	
Atezolizumab	10	5	5	
Durvalumab	3	2	1	
Treatment line of ICIs				
1st	15	10	5	<0.001
2nd	9	4	5	
≥3rd	31	3	28	
Bone‐modifying agents				
Yes	35	12	23	0.555
Denosumab	29	9	20	
Zoledronic acid	6	3	3	
No	20	5	15	
ECOG PS				
0–1	33	11	22	0.769
2–4	22	6	16	
Katagiri score				
4–6 points	32	13	19	0.098
7–10 points	23	4	19	
Metastasis to another organ				
Brain	15	4	11	0.557
Liver	12	3	9	
Others	19	3	16	
None	9	7	2	
Bone metastasis				
Monostotic	12	3	9	0.735
Multiple	43	14	29	
WBC ≥8600/mm^3^	12	1	11	0.079
ANC ≥6300/mm^3^	13	1	12	0.045
ALC ≤1000/mm^3^	25	9	16	0.562
NLR ≥2.1	43	10	33	0.033
Hemoglobin ≤12.7 g/dL	41	11	30	0.332
Platelet ≤158,000/mm^3^	4	1	3	0.808
PLR ≥197	40	10	30	0.189
LDH ≥222 U/L	29	8	21	0.771
CRP ≥0.14 mg/dL	44	10	34	0.024
Creatinine ≥0.94 mg/dL	6	1	5	0.654
Albumin ≤4.0 g/dL	43	11	32	0.158
ALP ≥322 U/L	20	4	16	0.235
CEA ≥5.0 ng/mL	40	12	28	0.745
CYFRA ≥3.5 ng/mL	36	10	26	0.187
KL‐6 ≥ 458 U/mL	26	8	18	0.863

Abbreviations: ALC, absolute lymphocyte count; ANC, absolute neutrophil count; CR, complete response; ECOG PS, Eastern Cooperative Oncology Group performance status; ICI, immune checkpoint inhibitor; NLR, neutrophil‐to‐lymphocyte ratio; PD, progressive disease; PLR, platelet‐to‐lymphocyte ratio; PR, partial response; SD, stable disease; TPS, tumor proportion score.

### 3.2 Therapeutic effect of ICIs

The rate of ICI response in patients with bone metastasis was 30.9%, with CR in three cases and PR in 14 cases. In contrast, 38 patients were included in the non‐responder group. In the responder group, pembrolizumab was the most commonly used ICI, and in most cases, ICIs were used as the first‐line therapy. Although the concomitant use of a BMA was not significantly different between the groups, it was more frequent in the responder group (70.6% vs. 60.5%, *p* = 0.55). Among the cases where a BMA was used, denosumab was the most common (82.9%). Moreover, in all cases with CR, ICIs, and denosumab were concomitantly administered. In the pretreatment blood sampling data, the inflammatory markers ANC, NLR, and CRP level were significantly lower in the responder group than in the non‐responder group (Table 1).

**FIGURE 1:**
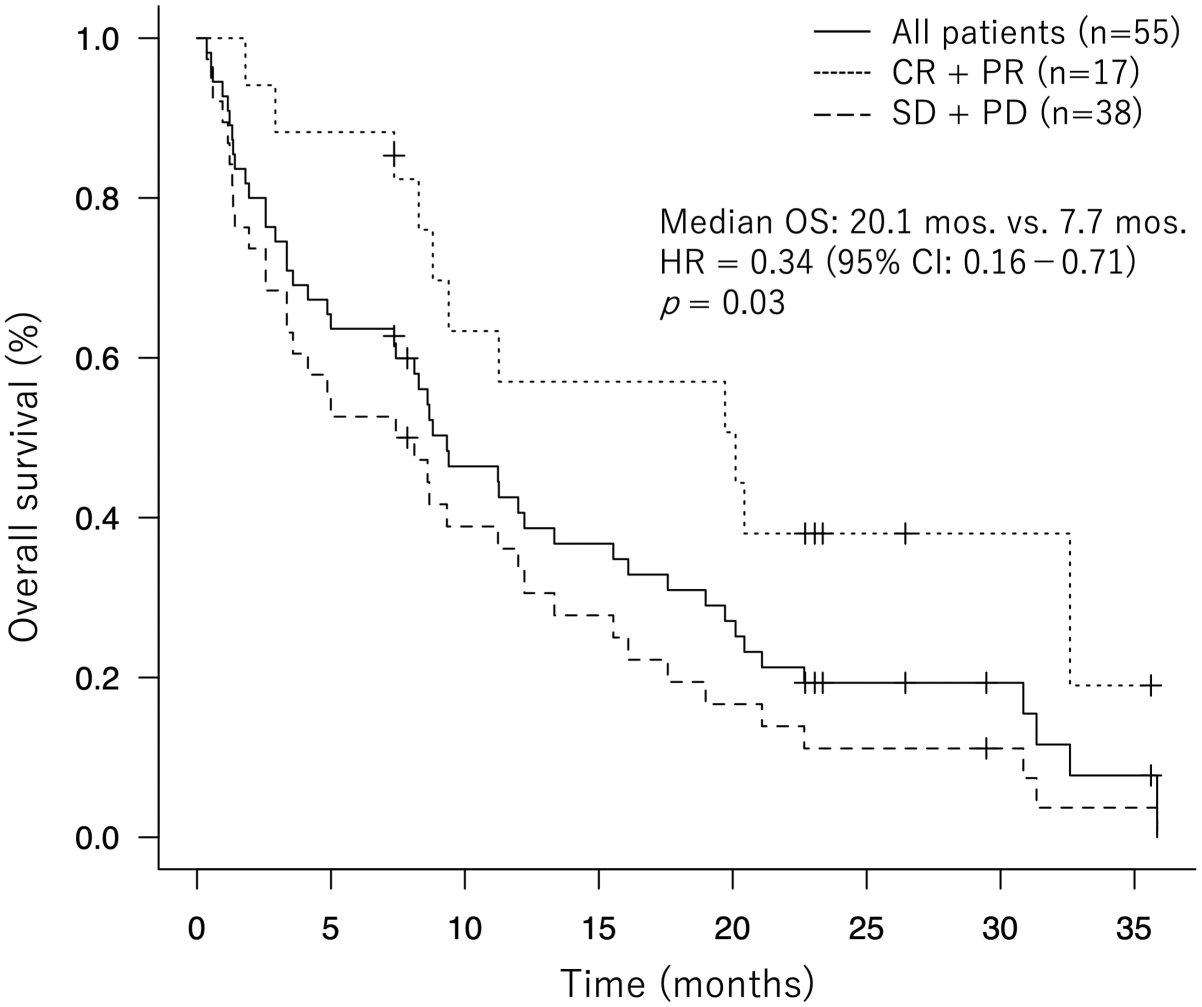
Overall survival rates of all patients, responders (CR + PR), and non‐responders (SD + PD).

The median survival time for all patients was 9.3 months, and the 1‐year and 2‐year survival rates were 40.6% and 19.3%, respectively. Furthermore, OS was significantly longer in the responder group than in the non‐responder group (20.1 months vs. 7.7 months; *p* = 0.03) (Figure 1). Katagiri score at the time of diagnosis with bone metastasis was 4–6 points in 32 cases and 7–10 points in 23 cases. The results of OS rate from the diagnosis of bone metastasis in this study are summarized in Table 2; notably, OS rates in this study were longer than those predicted by Katagiri et al.^30^

**TABLE 2:** Comparison of the overall survival rates using the Katagiri score.

	Katagiri score			
	4–6 points		7–10 points	
	Katagiri's prediction	Our study (*n* = 32)	Katagiri's prediction	Our study (*n* = 23)
6 months	74.0%	97.0%	26.9%	61.8%
12 months	49.3%	93.9%	6.0%	33.3%
24 months	27.6%	69.1%	2.1%	23.8%

### 3.3 Predictors of therapeutic response and prognosis

According to univariate analysis of variables associated with response to ICI treatment, there were significant differences observed between responders and non‐responders with regard to sex (male vs. female; *p* < 0.01), line of ICI therapy (1st vs. ≥2nd; *p* < 0.01), NLR (≥2.1 vs. <2.1; *p* = 0.02), and CRP (≥0.14 vs. <0.14; *p* = 0.01). Multivariate analysis of these variables identified the predictors of favorable response to ICI therapy as follows; female sex (*p* = 0.03), use of ICIs as first‐line therapy (*p* < 0.01), and NLR <2.1 (*p* = 0.03) (Table 3). Univariate analysis of variables associated with prognosis indicated that concomitant BMA use (yes vs. no: *p* = 0.02), Katagiri score (0–6 points vs. ≥7 points; *p* < 0.01), WBC (≥8600 /mm^3^ vs. <8600 /mm^3^; *p* = 0.03), ANC (≥6300 /mm^3^ vs. <6300 /mm^3^; *p* = 0.03), NLR (≥2.1 vs. <2.1; *p* < 0.02), CRP (≥0.14 mg/dL vs. <0.14 mg/dL; *p* = 0.02), and ALP (≥322 U/L vs. <322 U/L; *p* = 0.02) exhibited significant effects. Among them, five variables with low *p*‐values, which were considered to be significantly correlated with prognosis, were selected for the multivariate analysis owing to the limited number of patients. The multivariate analysis of these selected variables identified the predictors of good prognosis as follows; concomitant BMA use (*p* < 0.01), Katagiri score ≤6 points (*p* < 0.01), and NLR <2.1 (*p* = 0.02) (Table 4). Only pretreatment NLR predicted both therapeutic response and prognosis in advanced NSCLC patients with bone metastases who received ICI treatment.

**TABLE 3:** Univariate and multivariate logistic regression analyses to determine predictors of the therapeutic effect of ICIs on bone metastases.

		Univariate analysis		Multivariate analysis	
		OR (95% CI)	*p*‐value	OR (95% CI)	*p*‐value
Sex	Female	6.00 (1.65–21.80)	<0.01	7.62 (1.18–49.30)	0.03
Age	<70	2.76 (0.85–9.01)	0.09		
Histology	ADC	0.37 (0.07–1.93)	0.24		
Driver‐gene mutation	No	1.48 (0.42–5.26)	0.54		
PD‐L1 TPS	≥50%	1.33 (0.27–6.61)	0.72		
ICIs	Pemb vs. Others	1.25 (0.40–3.93)	0.70		
	Atezo vs. Others	2.75 (0.68–11.20)	0.15		
Treatment line of ICIs	1st	0.11 (0.03–0.41)	<0.01	0.05 (0.01–0.38)	<0.01
Bone‐modifying agents	Yes	1.57 (0.46–5.35)	0.47		
ECOG PS	0–1	0.75 (0.23–2.45)	0.63		
Katagiri score	0–6	0.31 (0.08–1.12)	0.07		
Metastasis to another organ	No	0.66 (0.21–2.09)	0.47		
Bone metastasis	Mono	1.45 (0.34–6.20)	0.61		
WBC	CO: ≥8600/mm^3^	0.15 (0.02–1.30)	0.08		
Neutrophil	CO: ≥6300/mm^3^	0.13 (0.02–1.14)	0.06		
Lymphocyte	CO: ≤1000/mm^3^	1.55 (0.49–4.88)	0.45		
NLR	CO: ≥2.1	0.21 (0.06–0.83)	0.02	0.11 (0.01–0.86)	0.03
Hemoglobin	CO: ≤12.7 g/dL	0.48 (0.14–1.73)	0.26		
Platelet	CO: ≤158,000/mm^3^	0.72 (0.07–7.56)	0.79		
PLR	CO: ≥197	0.38 (0.11–1.32)	0.12		
LDH	CO: ≥222 U/L	0.72 (0.23–2.27)	0.57		
CRP	CO: ≥0.14 mg/dL	0.17 (0.04–0.72)	0.01	0.26 (0.04–1.88)	0.18
Creatinine	CO: ≥0.94 mg/dL	0.41 (0.04–3.83)	0.43		
Albumin	CO: ≤4.0 g/dL	0.34 (0.09–1.29)	0.11		
ALP	CO: ≥322 U/L	0.42 (0.12–1.54)	0.19		
CEA	CO: ≥5.0 ng/mL	0.77 (0.21–2.79)	0.69		
CYFRA	CO: ≥3.5 ng/mL	0.38 (0.11–1.38)	0.14		
KL‐6	CO: ≥458 U/mL	0.88 (0.26–3.05)	0.85		

Abbreviations: ADC, adenocarcinoma; CI, confidence interval; CO, cutoff value; ECOG PS, Eastern Cooperative Oncology Group performance status; ICI, immune checkpoint inhibitor; NLR, neutrophil‐to‐lymphocyte ratio; OR, odds ratio; PLR, platelet‐to‐lymphocyte ratio; TPS, tumor proportion score.

**TABLE 4:** Univariate and multivariate Cox proportional hazards regression analyses to determine predictors of prognosis in NSCLC patients with bone metastasis.

		Median OS (95% CI)	Univariate analysis	Multivariate analysis	HR (95% CI)	*p*‐value
			HR (95% CI)	*p*‐value		
Sex	M	8.60 (4.86–15.54)	0.80 (0.42–1.57)	0.53		
	F	19.71 (1.81–21.09)				
Age	<70	8.11 (3.35–16.10)	0.65 (0.34–1.23)	0.19		
	≥70	11.99 (7.43–32.59)				
Histology	ADC	11.99 (4.86–18.99)	1.28 (0.62–2.61)	0.49		
	Non‐ADC	8.36 (1.31–12.22)				
Driver‐gene mutation	Yes	7.42 (0.36‐NA)	1.83 (0.78–4.23)	0.15		
	No	11.23 (4.86–18.98)				
PD‐L1 TPS	<49%	8.27 (1.35–19.71)	0.66 (0.27–1.64)	0.37		
	≥50%	9.06 (0.59–32.59)				
ICIs	Nivolumab	11.99 (1.41–18.99)	0.85 (0.60–1.22)	0.39		
	Pembrolizumab	8.27 (2.56–16.10)				
	Atezolizumab	19.71 (1.31‐NA)				
	Durvalumab	20.43 (8.11‐NA)				
Treatment line of ICIs	1st	18.11 (3.35‐NA)	1.98 (0.919–4.27)	0.06		
	≥2nd	8.61 (3.58–13.34)				
Bone‐modifying agents	Yes	15.34 (7.43–21.09)	2.03 (1.07–3.85)	0.02	2.95 (1.48–5.89)	<0.01
	No	6.49 (1.94–11.99)				
ECOG PS	0–1	16.54 (8.61–20.11)	1.73 (0.94–3.20)	0.07		
	2–4	4.93 (1.41–8.80)				
Katagiri score	0–6	16.10 (9.33–30.85)	2.92 (1.57–5.46)	<0.01	3.09 (1.53–6.23)	<0.01
	7–10	3.58 (1.81–8.11)				
Another metastasis	Yes	4.86 (2.56–17.58)	1.27 (0.70–2.34)	0.42		
	No	12.78 (8.61–20.44)				
Bone metastasis	Monostotic	18.11 (8.11‐NA)	1.97 (0.93–4.69)	0.06		
	Multiple	8.28 (3.35–13.34)				
WBC	<8600/mm^3^	10.22 (7.61–18.11)	2.30 (1.67–5.53)	0.03		
	≥8600/mm^3^	2.25 (0.53–8.28)				
ANC	<6300/mm^3^	11.34 (7.11–18.11)	2.23 (1.66–5.32)	0.03		
	≥6300/mm^3^	2.56 (0.59–8.28)				
ALC	>1000/mm^3^	8.67 (3.58–17.58)	0.95 (0.52–1.71)	0.85		
	≤1000/mm^3^	12.22 (4.14–20.11)				
NLR	<2.1	18.99 (8.80‐NA)	2.49 (1.05–5.91)	0.02	2.79 (1.11–7.00)	0.02
	≥2.1	8.11 (3.35–13.34)				
Hemoglobin	≤12.7 g/dL	8.11 (3.35–13.34)	2.16 (0.99–4.66)	0.06		
	>12.7 g/dL	20.44 (8.61‐NA)				
Platelet	≤158,000/mm^3^	13.34 (8.11‐NA)	0.89 (0.27–2.92)	0.85		
	>158,000/mm^3^	8.80 (4.14–16.10)				
PLR	<197	18.44 (8.11–32.59)	2.16 (0.99–4.66)	0.06		
	≥197	7.43 (3.35–11.24)				
LDH	≥222 U/L	4.14 (1.81–8.67)	1.85 (0.99–3.45)	0.06		
	<222 U/L	16.10 (11.99–20.44)				
CRP	<0.14 mg/dL	19.71 (13.34‐NA)	2.85 (1.19–6.84)	0.02	1.77 (0.66–4.74)	0.25
	≥0.14 mg/dL	8.11 (3.35–11.24)				
Creatinine	≥0.94 mg/dL	13.77 (1.21‐NA)	0.89 (0.35–2.29)	0.81		
	<0.94 mg/dL	8.80 (4.99–17.58)				
Albumin	≤4.0 g/dL	8.11 (3.35–12.22)	2.06 (0.95–4.45)	0.07		
	>4.0 g/dL	19.71 (8.61‐NA)				
ALP	<322 U/L	12.22 (8.28–21.09)	2.49 (1.20–3.98)	0.02	1.87 (0.85–3.78)	0.21
	≥322 U/L	5.36 (1.35–13.34)				
CEA	≥5.0 ng/mL	8.28 (3.58–13.34)	1.97 (0.95–4.06)	0.07		
	<5.0 ng/mL	16.10 (4.99–32.59)				
CYFRA	≥3.5 ng/mL	8.44 (3.58–13.34)	1.78 (0.82–3.86)	0.15		
	<3.5 ng/mL	16.10 (8.11‐NA)				
KL‐6	≥458 U/mL	8.08 (3.35–18.99)	1.21 (0.63–2.31)	0.57		
	<458 U/mL	13.34 (4.14–30.85)				

Abbreviations: ADC, adenocarcinoma; ALC, absolute lymphocyte count; ANC, absolute neutrophil count; CI, confidence interval; CR, complete response; ECOG PS, Eastern Cooperative Oncology Group performance status; F, female; HR, hazard ratio; ICI, immune checkpoint inhibitor; M, male; NLR, neutrophil‐to‐lymphocyte ratio; NLR, neutrophil‐to‐lymphocyte ratio; OS, overall survival; PD, progressive disease; PLR, platelet‐to‐lymphocyte ratio; PLR, platelet‐to‐lymphocyte ratio; PR, partial response; SD, stable disease; TPS, tumor proportion score.

## 4 DISCUSSION

This study is an extension of our previous analysis of the therapeutic effect of ICIs in NSCLC patients with bone metastases,^24^ and revealed novel predictors of favorable therapeutic response and prognosis in these patients. The response rate was 30.9%, and bone metastases completely disappeared (MDA criteria; CR) in three cases. Patients treated with ICIs showed better OS than would be expected from their Katagiri scores, the metric generally used to determine prognosis for cancer patients with bone metastases. First‐line treatment with pembrolizumab may have the strongest therapeutic effects. Female sex, use of ICIs as first‐line treatment, and NLR <2.1 predicted a favorable response to ICI treatment, whereas concomitant BMA use, Katagiri score ≤6 points, and NLR <2.1 predicted good prognosis. Pretreatment NLR was the only variable that predicted both ICI treatment response and prognosis.

Recent advances in ICI treatment have dramatically improved the clinical outcomes in various cancers and led to a paradigm shift in cancer treatment.^17^, ^18^ In clinical trials under several conditions, PD‐1 and PD‐L1 inhibitors have significantly improved prognostic indicators (OS and PFS) in advanced NSCLC compared with that of conventional anticancer drugs.^19^, ^20^, ^21^, ^22^ The response rates of nivolumab and pembrolizumab monotherapy for primary lung lesions have been reported as 19–20% and 44.8%, respectively,^20^, ^21^, ^22^ with favorable therapeutic effects on the primary lesion and prognosis of NSCLC. However, few reports on the outcomes of ICI treatment in patients with bone metastases are available, and the therapeutic effects remain incompletely understood.

Based on two cases of advanced NSCLC, in which the primary lung lesion and impending fracture of the lower extremity were dramatically ameliorated by pembrolizumab treatment,^23^ we previously summarized the therapeutic effects of ICIs on bone metastases and reported favorable clinical outcomes.^24^ To our knowledge, the only similar report is that by Nakata et al., who reported a 40% response rate to nivolumab monotherapy among 15 NSCLC patients with bone metastases.^33^ Although there was a difference in sample size, the response rates in our study were almost the same, supporting the notion that ICIs have favorable therapeutic effects on bone metastases, as well as primary lung lesions.

Moreover, our previous study showed that the OS of the group of patients with bone metastatic lesions who responded to ICI treatment was significantly prolonged than that of the non‐responsive group.^24^ This study included a larger number of patients than that of the previous studies and, similarly to the previous studies, showed significantly prolonged OS in the responder group (Figure 1). Similarly, Nakata et al.^33^ and De Giglio et al.^34^ reported that in NSCLC patients with bone metastases treated with ICIs, an early response, as determined by MDA criteria, is predictive of a good prognosis. These results suggest that a favorable prognosis can be expected in cases where ICI response to bone metastasis is observed. Therefore, new predictors of response to bone metastasis and prognosis are needed to establish optimal treatment strategies including ICIs for NSCLC with bone metastasis. Although some studies reported immunohistochemical and genetic variables such as PD‐L1 expression^26^, ^27^ and TMB^28^ as predictors of the therapeutic effects of ICIs in NSCLC, the detection of these biomarkers can depend on whether sufficient tumor tissue is obtained and requires invasive procedures for sampling.^25^ Thus, better predictors that can evaluate the therapeutic response to ICIs in patients with bone metastases without invasive procedures are required.

In this study, female sex was a predictor of favorable response to ICI treatment. Sex has been reported to influence innate and adaptive immune responses, as well as the expression and function of PD‐L1 and PD‐1.^35^, ^36^, ^37^ It has been suggested that women may be more resistant to immunotherapy because they have a weaker tumor immunogenic response than men.^35^ Indeed, for various cancers, including NSCLC, some studies have reported that ICI monotherapy is more effective in men than in women.^19^, ^20^, ^35^, ^38^ Our study is the first to evaluate the therapeutic effects of ICIs against bone metastases based on sex, although it is limited by the relatively small sample size. Previous studies have revealed that the bone marrow plays a key role in regulating the immune response and influences response to immunotherapy.^12^, ^39^ Thus, the immune response to bone metastases may differ from the response to the primary tumor, which may have influenced a better response to ICI treatment observed in women in this study. However, this is merely a hypothesis and the mechanism remains to be clarified.

Currently, ICIs can be used as the first‐line treatment of driver‐gene‐mutation‐negative advanced NSCLC with PD‐L1 expression greater than 50%. Furthermore, even if PD‐L1 expression is less than 50%, they can be used in the combination with conventional anticancer drugs. In previous reports, the concomitant use of pembrolizumab or atezolizumab with those anticancer drugs has been associated with better therapeutic effects in advanced NSCLC.^40^, ^41^, ^42^, ^43^, ^44^ Our results showed that the use of ICIs as first‐line treatment was a predictor of therapeutic response, supporting these reports. Although some ICI regimens are available as first‐line treatments, no studies have compared them directly, and no data regarding the most optimal regimen are available. In this study, no significant difference between ICI types was observed; however, pembrolizumab may be expected to have the greatest therapeutic effect on bone metastases. Based on these results, ICIs, especially pembrolizumab, may be recommended as a first‐line treatment for advanced NSCLC with bone metastasis.

In our study, 70.6% of bone metastasis cases were concomitantly treated with a BMA, most often denosumab. In particular, all patients who experienced CR based on MDA criteria underwent combined treatment with ICIs and denosumab, implying that the concomitant use of denosumab may enhance the therapeutic effects of ICIs. Previous studies on advanced NSCLC have reported that clinical outcomes such as response rate, OS, and PFS were improved with the combination therapy of ICI and denosumab.^60^, ^61^ Similarly, Angela et al. reported that the concomitant use of a PD‐1 inhibitor and denosumab showed strong therapeutic effects on bone metastases in melanoma patients.^45^ Moreover, in mouse models of melanoma, prostate cancer, and colon cancer, concomitant use of PD‐1/PD‐L1 inhibitors and receptor activator of nuclear factor kappa‐B ligand (RANKL) inhibitors enhanced anti‐metastatic activity and suppressed subcutaneous tumor growth.^46^, ^47^ In recent years, the RANKL pathway has played an important role in anticancer therapies targeting bone and the immune system, and the development of ICI targeting the pathway has been considered as a way to improve the therapeutic effect of ICI.^48^ In this study, the concomitant use of ICIs and a BMA, such as denosumab, significantly prolonged OS time and was found to predict a favorable prognosis. Considering these results, the concomitant use of ICIs and a BMA, especially denosumab, is recommended for the treatment of advanced NSCLC with bone metastasis; however, the mechanism by which the combined treatment produces these antitumor effects remains to be clarified.

Previous studies have reported that inflammation is associated with prognosis in patients with solid tumors and plays a key role in tumor development and invasion.^25^, ^49^, ^50^, ^51^, ^52^ In clinical practice, blood sampling is the easiest and least invasive method of evaluating inflammation, with peripheral serum indicators, such as WBC and CRP, which are considered biomarkers of systemic inflammation, being correlated with prognosis and therapeutic effects in cancer patients.^49^, ^53^ For NSCLC patients undergoing ICI treatment, NLR, which is an indicator of systemic inflammation, has been suggested as a reliable hematological indicator of prognosis in ICI treatment,^49^, ^54^, ^55^, ^56^ and a high NLR is reportedly associated with a poor prognosis.^57^, ^58^, ^59^ Our results, similar to those of previous reports, showed that pretreatment NLR was the only predictor of both ICI treatment response and prognosis in advanced NSCLC patients with bone metastasis, making it a very important indicator. Bongiovanni et al.^62^ reported NLR as a useful prognostic marker in ICI administration to treat NSCLC with bone metastases, which was consistent with our results. Furthermore, Platini et al.^63^ reported the usefulness of NLR for predicting the prognosis of advanced NSCLC treated with immunotherapy in a systematic review and meta‐analysis. However, these previous studies reported that the reliable NLR cutoff value was 5, which is marginally different from our study. The reasons for this difference in the cutoff value can be due to the small sample size of this study and the exclusion of patients with infectious diseases or other causes of an elevated inflammatory response at the time of pretreatment blood sampling. Another reason could be a possible selection bias owing to the ICI treatment provided to patients in good general condition in this study.

With improvements in multidisciplinary therapy, including ICI treatment, the survival time of cancer patients has been prolonged, and the number of patients with bone metastasis may increase in the future. Therefore, understanding the therapeutic effects of ICIs, which are coming to play a central role in medication therapy, in NSCLC is crucial. The results of this study are expected to be useful for future treatments. In future studies, it will be necessary to increase the study population and conduct large‐scale analyses.

This study has limitations. First, this study was a retrospective study conducted in a single institution, with a relatively small number of cases. A multicenter study is necessary to evaluate a large number of cases. Second, this study did not consider treatment regimens combining an ICI with conventional anticancer drugs or with other ICIs, which have reportedly produced better therapeutic effects in clinical trials. Third, although the influence of comorbidities and concomitant medications on the therapeutic effect of ICIs is not clear, it was not investigated in this study. Few studies on the therapeutic effectiveness of ICIs in treating advanced NSCLC with bone metastases have been conducted, and this study probably indicates a trend toward novel factors that can predict the prognosis and clinical responses of ICIs in these patients. Further studies with larger sample sizes will be performed.

## 5 CONCLUSIONS

This study revealed that ICI treatment produced favorable therapeutic effects and improved prognosis in advanced NSCLC patients with bone metastasis and identified novel predictors for the treatment response and prognosis. Specifically, female sex, use of ICIs as first‐line treatment, and NLR <2.1 were independent predictors of favorable response to ICI treatment, whereas concomitant use of BMA, Katagiri score ≤6 points, and NLR <2.1 were independent predictors of a favorable prognosis. Pretreatment NLR can be considered the most important predictor of clinical outcomes in advanced NSCLC patients undergoing ICI treatment.

## AUTHOR CONTRIBUTIONS

**Yohei Asano:** Conceptualization (lead); data curation (lead); formal analysis (lead); investigation (lead); methodology (lead); project administration (equal); writing – original draft (lead). **Norio Yamamoto:** Data curation (supporting); formal analysis (supporting); investigation (supporting); writing – review and editing (lead). **Satoru Demura:** Data curation (supporting); formal analysis (supporting); investigation (supporting). **Katsuhiro Hayashi:** Data curation (supporting); formal analysis (supporting); investigation (supporting). **Akihiko Takeuchi:** Conceptualization (lead); data curation (equal); formal analysis (equal); investigation (equal); methodology (equal); project administration (lead); writing – review and editing (lead). **Satoshi Kato:** Data curation (supporting); formal analysis (supporting); investigation (supporting). **Shinji Miwa:** Data curation (supporting); formal analysis (supporting); investigation (supporting). **Kentaro Igarashi:** Data curation (supporting); formal analysis (supporting); investigation (supporting). **Takashi Higuchi:** Data curation (supporting); formal analysis (supporting); investigation (supporting). **Yuta Taniguchi:** Data curation (supporting); formal analysis (supporting); investigation (supporting). **Sei Morinaga:** Data curation (supporting); formal analysis (supporting); investigation (supporting). **Takashi Sone:** Conceptualization (supporting); data curation (equal); formal analysis (supporting); investigation (supporting); writing – review and editing (supporting). **Miho Okuda:** Data curation (supporting); investigation (supporting); project administration (supporting); writing – review and editing (supporting). **Isao Matsumoto:** Data curation (supporting); investigation (supporting); writing – review and editing (supporting). **Seiji Yano:** Data curation (supporting); investigation (supporting); writing – review and editing (supporting). **Hiroyuki Tsuchiya:** Conceptualization (equal); data curation (supporting); formal analysis (supporting); investigation (supporting); methodology (supporting); project administration (supporting); writing – review and editing (lead).

## FUNDING INFORMATION

Not applicable.

## CONFLICT OF INTEREST STATEMENT

The authors have no conflict of interest.

## ETHICS STATEMENT

This study was approved by the Institutional Reviewer Board of Kanazawa University Hospital (no. 2019–323), and informed consent was obtained from all subjects based on the provisions of the Declaration of Helsinki.

## Data Availability

The datasets used and/or analyzed during the current study are available from the corresponding author upon reasonable request.
